# Performance and emission evaluation of CI engine fueled with ethanol diesel emulsion using NiZnFe_2_O_4_ nanoparticle additive

**DOI:** 10.1016/j.heliyon.2022.e11639

**Published:** 2022-11-14

**Authors:** Deresse Firew, Ramesh Babu Nallamothu, Getachew Alemayehu, Rajendiran Gopal

**Affiliations:** a Mechanical Engineering Department, SoMCME, Adama Science and Technology University, Adama, Ethiopia; b Department of Motor Vehicle Engineering, Defense Engineering College, Bishoftu, Ethiopia

**Keywords:** Alternative fuels, Micro emulsion, Nanoparticle, Smoke, Performance

## Abstract

Diesel engines are widely used due to their higher thermal efficiency and lower Hydrocarbon (HC) emissions. The price fluctuation of petroleum fuel; the none-renewability and different hazardous emissions from petro-diesel, researchers are searching for a suitable renewable energy source for diesel engine. Ethanol is one of the promising alternative fuels that could be used to power diesel engine. Some of the techniques so far tried are ethanol fumigation, dual injection and blending. The former techniques need different engine modification to enable the additional systems to work on the existing engines. Blending is the best technique to substitute the existing diesel fuel as long as we prepare it as per the current diesel fuel minimum requirement. For this specific case micro emulsion of ethanol in diesel fuel with emulsifier is prepared by using magnetic stirrer and bath type ultrasonicator. Nickel Zinc Iron Oxide (NiZnFe_2_O_4_) nanoparticle is added in one of the selected emulsion which is having 10% ethanol in three different dose as additive to see the effect on the performance and emission variation of the emulsion compared with diesel fuel. The performance of the prepared sample fuels are tested on a test rig with single cylinder diesel engine. The emission is measured by using exhaust gas analyzer and smoke opacity meter. The specific fuel consumption, brake torque and brake power of E5 (95% diesel and 5% ethanol), E10 (90% diesel and 10% ethanol) and E15 (85% diesel and 15% ethanol) are evaluated and the result showed 27, 34 and 37% increment in fuel consumption; 15, 5 and 15% reduction in brake torque and the power reduction is 19, 7 and 12% respectively. E10 is selected for further test with addition of the nanoparticle with 25, 50 and 100ppm named as E103N25 (E10 with 25ppm NiZnFe_2_O_4_ nanoparticle), E103N50 (E10 with 50ppm NiZnFe_2_O_4_ nanoparticle) and E102N100 (E10 with 100 ppm NiZnFe_2_O_4_ nanoparticle). The result showed fuel consumption reduction by 16, 27 and 27% torque output increment of 1, 2 and 2% and power output increment of 5, 7 and 7% respectively compared with E10. The emission result revealed that the HC emission reduced by 50, 58 and 71% for E103N25, E103N50 and E103N100 fuel samples respectively, Carbon monoxide (CO) emission reduced by 67, 80 and 81% for E103N25, E103N50 and E103N100 fuel samples respectively. The reduction in smoke emission is reduced by 60, 69 and 61% for E103N25, E103N50 and E103N100 fuel samples respectively. Nitrogen oxide (NO_X_) and Carbon dioxide (CO_2_) emissions increased with the amount of nanoparticle added in to the fuel.

## 1 Introduction

Diesel engines are preferred for their high thermal efficiency and good drivability and are widely applied for heavy-duty machinery such as farm tractors, combine harvesters, electric generators, and light and heavy duty public transport vehicles [1]. The stringent emission regulations set by many countries, the fluctuating price due to socioeconomic inconsistency of countries, uneven distribution of the petroleum resource in the world and the suspicion of petroleum depletion in the future are forcing human beings to look for alternative fuel to substitute diesel fuel partly or totally [2]. Ethanol has been widely used to substitute gasoline fuel. However, due to low viscosity, cetane number, heating value and flash point temperature its use in diesel engines remained objectionable [3]. Researchers have kept on trying to utilize ethanol as a substitute for diesel fuel. The different techniques to use ethanol in diesel CI (compression ignition) engines are ethanol fumigation by carburetion or by port injection, multiple injection, and emulsion. The emulsion method could be used on existing engines without any modification [4].

For this specific research work, ethanol is considered as the driving range extender by substituting up to 15% volume percentage of the diesel fuel so that the amount of petro-diesel consumption necessary to power the engine could be reduced accordingly. One of the main problems with the utilization of ethanol diesel emulsion is its stability. The stability of ethanol diesel emulsion depends on the amount of water content in the base ethanol and ambient temperature. As the amount of water in the fuel is higher the tendency of separation also increases [5]. The ethanol used for this specific research is produced from molasses which is the byproduct of sugar production. The process of removing water from ethanol incurs an additional cost on the ethanol so the bioethanol obtained for the test is the one having a purity of 97%. The solution to maintaining its stability is to use an emulsifying agent. The emulsifier used to stabilize the solution is the mixture of Tween 80 and span 80 to obtain the proper hydrophilic-lipophilic balance (HLB) [6].

To improve the performance and exhaust emission characteristics of the emulsified fuel, Nickel Zinc Iron Oxide Nanoparticles (NiZnFe_2_O_4_) are doped in different ppm amount. To mix the nanoparticle with the prepared sample fuels homogeneously and maintain the nanoparticle to remain suspended in the fuel, a surfactant called, Cetyl Trimethyl Ammonium Bromide (CTAB) is used. Cetyl trimethyl ammonium bromide is an organic bromide salt of cetyl trimethyl ammonium, which functions as a detergent and a surfactant. It is an organic bromide salt of quaternary ammonium [7].

The addition of surfactant improves the stability of the nanoparticles added in to the fuel by converting the surfaces of nanoparticle, from hydrophobic to hydrophilic and vice versa. It also reduces the surface tension, coagulation and agglomeration by breaking the asymmetric bonds. The surfactant particle positions them and creates a point of continuity between the nanoparticle and the base fuel at the interface [8].

Nanoparticles, as a fuel additive, demonstrated a considerable improvement in the performance of diesel engine and reduced harmful exhaust gas emissions because of the nanoparticles' dispersion in the sample fuels. The nanoparticle added fuels have better thermophysical properties, particularly high thermal conductivity. However, the uniform dispersion of the nanoparticles in the base fuel and maintaining the stability is a major problem in the use of nanoparticles as fuel additives. This problem is overcome by the use of appropriate surfactant, which could enhance the stability of nanoparticle [9].

To improve the engine performance and reduce the pollutant emission of ethanol diesel blends, the use of additives is important. Nanoparticle additives are attracting the research community for fuel additive and many are working on improving the combustion characteristics, as a result improving the engine thermal efficiency and reducing exhaust pollutant gasses. There are many research reports conducted on the utilization of metallic nanoparticles such as cerium, iron, manganese, zinc and copper as fuel additives in different bio-fuels [10, 11].

To improve the engine performance and reduce exhaust emission, Sajith et al. conducted experimental research to see the physicochemical properties, performance and exhaust emission characteristics of biodiesel with cerium oxide nanoparticle additive. They applied 20, 40, 60, and 80ppm of nanoparticles to see their effect on the engine operating parameters. The results showed that the viscosity and flash point temperature increase with the amount of the cerium oxide nanoparticles increment. The values of Exhaust emission mainly NOx and UHC (unburned hydrocarbon) were reduced by 45% and 30%, respectively [12].

Harish Venu et al. prepared nanodiesel fuels by adding aluminum oxide nanoparticles in varying mass fractions and homogenized them using a mechanical stirrer and ultrasonicator. The Physicochemical Properties of the nanodiesel were tested and confirmed to run the engine after being compared with neat diesel. The engine performance and emission test results are concluded as there is improvement in the performance and a significant reduction in exhaust gas pollutant emission. The fuel with 25 ppm nanoparticle additive showed the best result and an ignition timing of 4° retarded from the initial ignition timing is also recommended. Advancing the injection timing lowered BSFC and smoke by 12.5% and 45.45% in part load condition however CO_2_ and NOx emission increased by 3.27% and 15.98% [13].

The influence of Zinc Oxide nanoparticles in Grape Seed biodiesel diesel blend fuel is studied by Elango (2014), on the performance and emission characteristics. The concentration of Zinc Oxide added to the blended fuel is 50 ppm and 100 ppm. The test is conducted on a four cylinder diesel engine. From the emission test result, they concluded that ZnO nanoparticle addition increased NOx and CO_2_ emissions, whereas the amount of CO, HC and smoke emissions have reduced [14].

Meshack Hawi et al. conducted an experiment on a four-stroke, single-cylinder, direct injection diesel engine using waste cooking Biodiesel diesel blend (B30) adding iron-doped cerium oxide (FeCeO2) nanoparticles. The nanoparticles were applied in the sample fuels at an amount of 90 ppm with the aid of an ultrasonicator. Tests were conducted at 2000 rpm constant engine speed varying loads from 0 to 12 Nm with neat diesel and biodiesel–diesel blends. Using neat diesel as the base fuel for reference the engine combustion, performance, and emission characteristics of the test fuel were compared. The test results indicated the peak cylinder pressure is improved by 3.5% due to nanoparticle addition. A NO_x_ reduction of up to 15.7% was recorded. Carbon monoxide was reduced by 15.7%. The brake thermal efficiency of the engine has increased by and the brake-specific fuel consumption is reduced [15].

Ethanol is the most widely produced biofuel in the world. It is in use as fuel for gasoline engines blending with gasoline fuel [16]. Problems like immiscibility, low calorific value, and low cetane number make ethanol utilization in diesel fuel difficult. To overcome the problems and to enable the use of ethanol in diesel engine methods such as emulsifying with diesel fuel and applying different additives to improve the physicochemical properties to meet the minimum requirements of diesel fuel is investigated by many researchers [17]. Metallic nanoparticles are some of the additives studied extensively. The effects of various single element nanoparticles in different fuels are studied to identify their consequence on diesel engine performance and emission characteristics [18]. However, Nickel Zinc Iron Oxide Nanoparticles (NiZnFe_2_O_4_) three elemental nanoparticles effect, in ethanol diesel emulsion is not reported so far.

The objective of this study is to investigate the effect of ethanol and Nickel Zinc Iron Oxide nanoparticles in ethanol diesel emulsion altering ethanol amount with 5, 10 and 15% by volume ratio and nanoparticle amount of 25, 50 and 100 ppm addition in E10 emulsion sample fuel to see the performance and emission characteristics in a diesel engine. The sample testing fuels are emulsified and the nanoparticle is dispersed in the prepared emulsion. The fuel parameters of the sample fuels were obtained. The compositions and fuel parameters of the emulsified fuels are compared with standard diesel fuel properties to see the possibility to run on the selected engine.

The aim of this research is to replace as much petro-diesel as practicable with locally produced renewable bioethanol in order to increase the driving range per unit of petro-diesel fuel and reduce hazardous emissions from diesel engine exhaust by encouraging oxygenated fuel combustion.

## 2 Methodology

**Figure 1:**
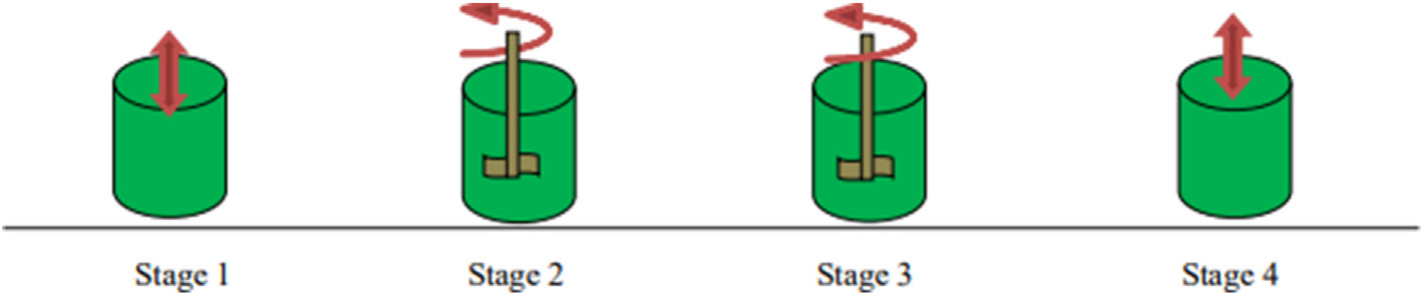
Stage by stage emulsion preparation process.

Diesel fuel and ethanol are prepared and different emulsions are prepared in three different proportions by varying the amount of ethanol by volume percentage. The ethanol used for this research work is aqueous ethanol which is having a water content of 3%. Maintain the stability of the diesel ethanol emulsion emulsifying agent having an HLB (hydrophilic-lipophilic balance) of 9 is prepared by mixing two different emulsifying agents namely tween80 and span80. Tween80 is an emulsifier having a high HLB value of 15 and span80 has a low HLB of 4.3 [19]. To prepare an emulsifying solution with the desired value of HLB between 4.3 and 15 is to mix the two emulsifiers in the correct proportion. To gate a solution with HLB of 9 the formula in Eq. (1) is applied. From the competition result, the proportion required in mass percentage is 44% of Tween80 and 66% of Span80. The mixing of the emulsifier is performed with an ultrasonicator at room temperature of 30 °C and a frequency of 40 kHz by operating the machine for the duty cycle indicated in Table 1 as shown in Figure 1 [20].

(1) $$\text{HLB}\;\text{of}\;\text{Emulsifier}\;\text{Mixture}=\frac{\text{Mass}\;\text{Span}\;80\times \text{HLB}\;\text{Span}\;80+\text{Mass}\;\text{tween}\;80\times \text{HLB}\;\text{Tween}\;80}{\text{MassTween}\;80+\text{MassSpan}\;80}\;$$

$$\text{mass}\;\%\text{Tween}80=\frac{\text{HLB}\;\text{of}\;\text{Mixture}-\text{HLB}\;\text{of}\;\text{Span}\;80}{\text{HLB}\;\text{of}\;\text{Tween}\;80-\text{HLB}\;\text{of}\;\text{Span}\;80}$$

$$\text{mass}\;\%\text{Tween}80=\frac{9-4.3}{15.0-4.3}=0.44=44\%$$

mass%Span80=100%−44%=66%

**Table 1:** Operating conditions for emulsified fuels preparation.

Stage	Process	factors	Composition
1st	Sonication	25 °C, 40 kHz, 30 min	Tween 80 + Span 80
2nd	Magnatic Steering	25 °C, 1,500 rpm, 20 min	Diesel + ethanol + emulsifier
3rd	Magnatic Steering	25 °C, 1,500 rpm, 10 min	Emulsion + nanoparticle + surfactant
4th	Sonication	25 °C, 40 kHz, 30 min	Emulsion + nanoparticle + surfactant

The emulsion process is done by mixing ethanol and diesel with a predetermined proportion and 2% of the emulsifier by volume is applied to enhance the emulsification and keep the solution stay homogeneous for a longer period of time. A magnetic stirrer is used as a mechanical agitator to mix the components together for about 20 min at room temperature. Nickel Zinc Iron Oxide Nanoparticles (NiZnFe_2_O_4_) is applied to the emulsion in the amount of 25, 50 and 100 ppm, and Cetyl Trimethyl Ammonium Bromide (CTAB) of the same amount in mass is used as a surfactant to protect the nanoparticle from sediment at the bottom of the emulsion and make dispersed throughout the solution. After adding the nanoparticle and the surfactant for 10 more minutes it is steered. Finally, to ensure the micro emulsion of the fuel constituents it is once again shacked with an ultrasonicator for 30 min and the fuel is made ready for the test [21].

**Figure 2:**
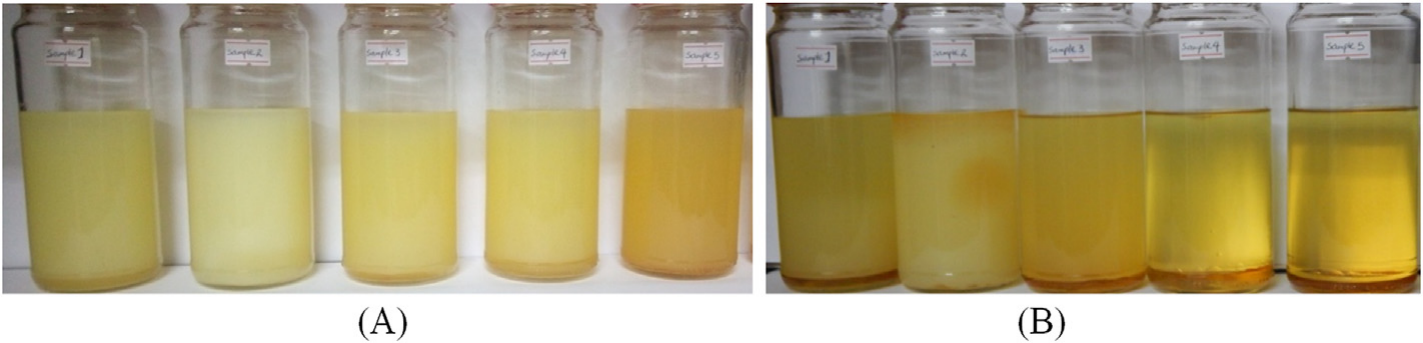
Sample fuels prepared for inspection, (A) before separation; (B) after separation.

Five samples are chosen from a list of fuels produced to analyze performance and emission characteristics to determine the emulsion stability of the test fuels. The stability of emulsified fuels in various proportions is tested at room temperature, which varies from 20 to 30 °C depending on the ambient weather conditions. As illustrated in Figure 2(A) which is taken right after the emulsion is made as the sample fuels in Table 2 are kept in a closed cup transparent glass container to visually evaluate the ethanol condition and nanoparticle stability. The inspection takes place every 30 min for the first 4 h, then every hour for the next 4 h, then every 12 h for the next nineteen days. Figure 2(B) shows the status of separation of the sample fuel after nineteen days.

**Table 2:** List of fuel samples for stability test.

Fuel sample	Fuel mixture	Surfactant	Emulsifier
D95E5	95% Diedel + 5% Ethanol		Tween 80, HLB = 15 and span 80, HLB = 4.3
D90E10	90% Diedel + 10% ethanol		44% tween
D85E15	85% Diedel + 15% ethanol		56% span
D90E103N50	90% Diedel + 10% Ethanol + 50 ppm NP	Cetyl Trimethyl Ammonium Bromide (CTAB)	About 2%
D90E10 3N100	90% Diedel +10% Ethanol + 100 ppm NP		

**Figure 3:**
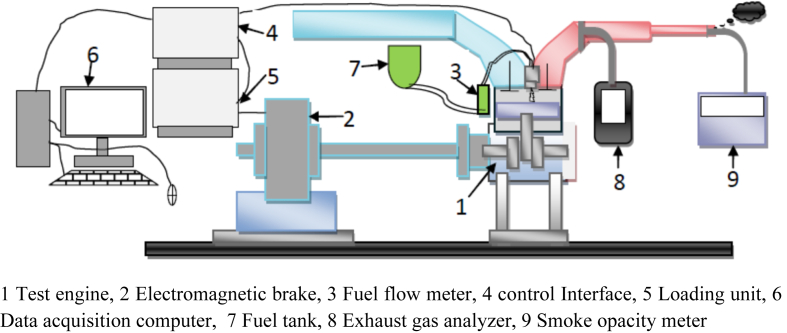
Performance and emission test setup.

The performance and emission characteristics are practically tested using a single-cylinder engine test rig with an eddy current type dynamometer, loading unit, control interface, computer and data acquisition system. Exhaust gas emission characteristics of the fuels are evaluated with an exhaust gas analyzer and smoke opacity meter. The arrangement of the performance and emission test is shown in Figure 3.

### 2.1 Performance evaluation

**Figure 4:**
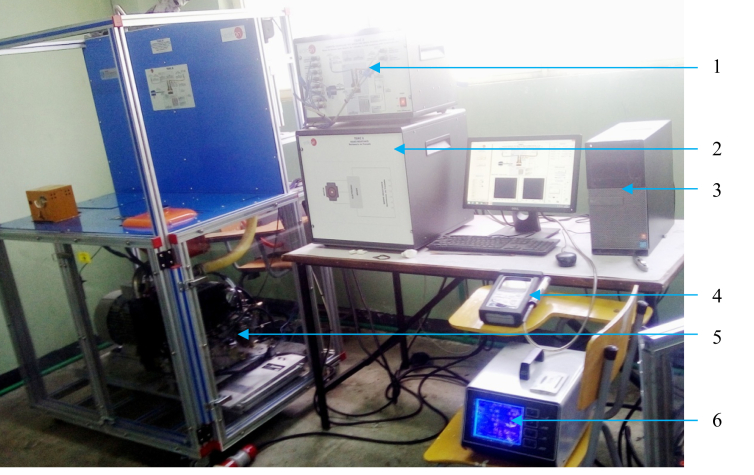
Materials used in the laboratory includes, 1 – Control interface, 2 – Loading unit, 3 – Data acquisition and management computer, 4 – Exhaust gas analyzer, 5 – Engine with dynamometer, 6 – Smoke meter.

The engine performance characteristics using the sample fuel is tested using an engine test rig. The test rig is made with a single cylinder engine and eddy current type dynamometer. Laboratory utensils such as engine dynamometer, smoke meter and exhaust gas analyzer used are shown in Figure 4. Single-cylinder four-stroke diesel engine is used for the test and the specification of the test engine is given in Table 3. The engine performance testing is carried out in Addis Ababa science and Technology University (AASTU), Mechanical Engineering department.

**Table 3:** Specification of the test engine.

Make/model	Kubota corporation, E300-ES01
Engine type	Single-cylinder, water-cooled, 4-stroke diesel engine
Bore-stroke	77.0 mm × 70.0 mm
Compression ratio	24:1
Displacement	309 cc
Rated power	5.2 kW @ 3000 r/min (rpm)
Bare idle speed	900–1000 rpm
Injecting Timing	27^0^ BTDC
Injection pump	Bosch K type mini pump
Injection pressure	140 kgf/cm^2^

The test is started by operating the engine for about 30 min to reach to its operating temperature. After the engine is warmed up the performance parameters of diesel fuel is recorded as a baseline data. Before operating the engine with the other fuel samples the former fuel in the fuel system is fully drained and the engine is allowed to run for additional 30 min until the remaining fuel clears out from the fuel liens and the exhaust gas measuring devises are setup. The same procedure is followed for each fuel sample. The test rig is designed to measure the performance parameter by varying the engine load so load condition is varied using the data acquisition and management computer to increase or decrease the load on the loading unit. Power and torque values are recorded by the data acquisition system with a click on the desired instant. The test value is recorded by varying the load from 0% to 80% with 10% difference. The test result is registered three times to reduce uncertainty and average value is used as a final value.

### 2.2 Emission evaluation

#### 2.2.1 Exhaust gas measurement

The exhaust gas measurement is started first by Calibrating the automatic zero during this sequence the analyzer pumps fresh air into the sensors to allow them to zero and the oxygen sensor to be set to 20.9 %. Once the initialization is completed the analyzer sets the CO, HC, CO_2_ and NO sensors to zero if fitted and set oxygen to 20.9% when the exhaust sampling probe of the analyzer is kept to pump in fresh air. Then the sample gas collector probe is properly connected to the end of the exhaust pipe. And measurement is taken for each load variation, allowing 1 min measurement settling time. CO_2_, CO, O_2_, HC, NO and λ measurements are recorded three times and the average value is taken for each gas value.

#### 2.2.2 Smoke opacity check

Variation of the Smoke density is measured by using opacity meter. The sample collecting probe is attached to the end of the exhaust pipe according to the opacimeter manual. The opacimeter is sustained to run for about 30 min to warm up and zero calibration. After the instrument is calibrated the level opacity is measured according to the measuring scale.

Exhaust gas analyzer is used to measure the amount of CO, CO_2_, HC, O_2_ NO_x_ and oxygen balance (Lambda) in the exhaust gas. The specification of the exhaust gas analyzer is given in Table 4.

**Table 4:** Technical data of the exhaust gas analyzer.

ITEM	Measurement Range	Resolution
CO	(0–10%),	0.01
CO_2_	(0–16%),	0.1
HC	(0–5000 ppm),	1
O_2_	(0–21%),	0.01
NOx	(0–5000 ppm)	1
Lambda	(0.8–1.2)	

#### 2.2.3 Smoke meter/Opacimeter

Intensity of smoke emitted from the exhaust is measured by smoke meter/opacimeter. It measures the smoke density within the range of 0–99.90% opacity with a resolution of 0.01% as indicated in the specification of the meter indicated in Table 5.

**Table 5:** Technical Data of the smoke meter/opacimeter

ITEM	Resolution	Measurement Range	Absolute Accuracy	Relative Accuracy
Opacity(N)	0.01%	0–99.90%	±2%	
Optical Absorption Coefficient(K)	0.01 m^−1^	0–16.08 m^−1^		0.05 m^−1^
Oil Temperature	1 °C	0–200 °C	±5 °C	
Gas Temperature	1 °C	0–150 °C	±5 °C	

## 3 Result and discussion

### 3.1 Fuel stability

**Figure 5:**
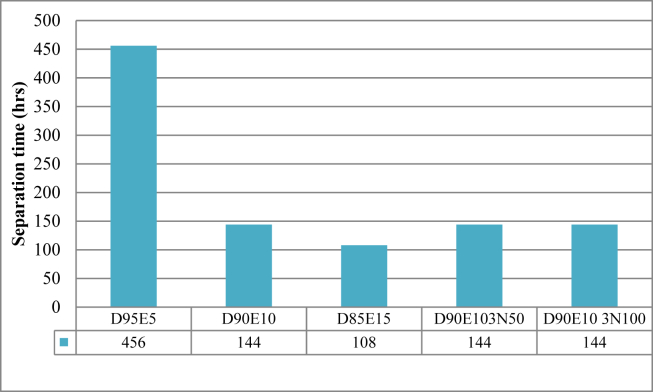
The influence of ethanol content on the stability of the emulsion.

The monitoring value of the emulsion stability showed that the nanoparticle added in to the emulsion remained suspended for four days. After the fourth day, all the nanoparticle added into the fuel sediment in the bottom of the inspection glass, in line with Mohammed S et al. [22]. When the condition of the ethanol separation from the diesel fuel is seen, the D95E5 fuel sample took about 19 days to separate. The next fuel sample, D90E10, D90E103N50 and D90E10 3N100 stayed only for 144 h without separating. The sample fuel D85E15 stayed the shortest time, 108 h without separating. The evaluated result indicated the amount of ethanol in the emulsified fuel highly affects the stability of the emulsion. As the amount of ethanol increases the tendency of separation also increase due to the physical property such as density difference of ethanol and diesel fuel and the water contained in the ethanol in line with Ghannam, M. et al. [23]. Figure 5 shows the time the emulsified fuels took to separate.

### 3.2 Performance

The performance of the sample fuel is evaluated by using an engine test rig. The brake specific fuel consumption, brake torque and brake power are measured. The measurement is done by varying the load condition from 0 to 80% with interval of 10% load. The characteristics of each performance parameters are discussed as indicated in the following paragraphs.

#### 3.2.1 Brake specific fuel consumption

**Figure 6:**
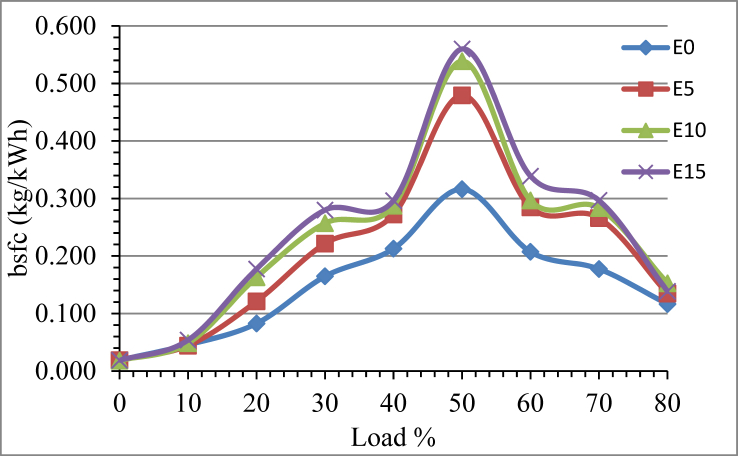
The effect of ethanol on engine fuel consumption.

**Figure 7:**
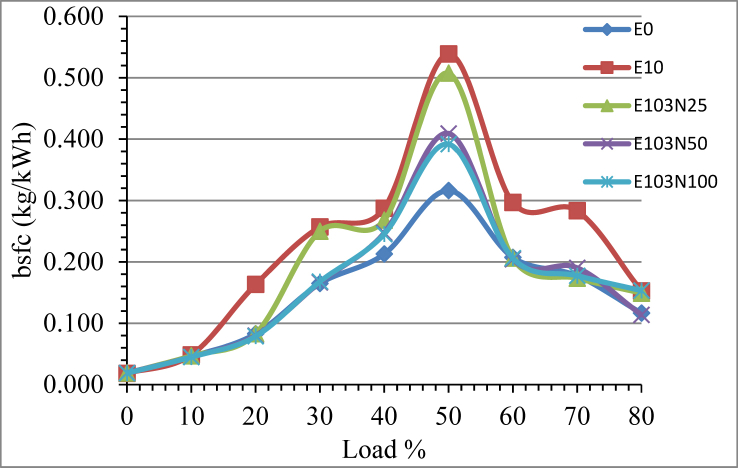
The effect of Nickel Zinc Iron Oxide (NiZnFe_2_O_4_) adding in E10 on engine fuel consumption.

The fuel measurement result revealed that the brake specific fuel consumption pattern is the same for all the sample fuels from 0% to 80% load with 10% variation. At low load condition the engine runs at high speed, this makes injection pump governor to set the control rack to the minimum fuel delivery volume position. The bsfc is small at low load condition due to the power required is small so the fuel delivery is restricted. Up to 50% load increment the fuel consumption also increases due to the speed of the engine decreases, resulting in the fuel pump to increase the fuel pump volume to increase. The bsfc start to reduce as we raise the engine load from 50% to 80% as indicated in Figures 6 and 7. Beyond 80% load, the engine hesitates to run vibrating harshly and the test is limited to a maximum loading of 80%. The reduced bsfc at high engine load is due to improved combustion. As the engine speed decreases, the fuel gates sufficient time to complete the combustion process resulting in increment the engine combustion temperature and in cylinder pressure. As shown in Figure 3 in diesel ethanol emulsion the amount of ethanol greatly affected the bsfc. The cetane number and calorific value which highly influences the combustion efficiency reduces as ethanol amount increased. The data collected from test showed 27.17, 34.39 and 37.87 percent bsfc rises for E5, E10 and E15 fuel samples respectively in line with Madhavi S [24].

Nickel Zinc Iron Oxide (NiZnFe_2_O_4_) Nanoparticles bsfc effect on E10 is plotted on the graph in Figure 7. The addition of nanoparticle reduced the fuel consumption of E10 emulsion prepared as E103N25, E103N50 and E103N100 by 16.79, 27.86 and 27.48% respectively when compared with E10 without nanoparticle addition. Compared to pure diesel, the fuel consumption of the nanoparticle added fuel samples show increment. Nanoparticles have catalytic properties to enhance combustion reaction, high thermal conductivity, and increased surface area to volume ratio which leads to improved heat release rate resulting cylinder pressure and temperature increment. Improved fuel combustion results in high power output leading in fuel consumption reduction.

#### 3.2.2 Brake torque

**Figure 8:**
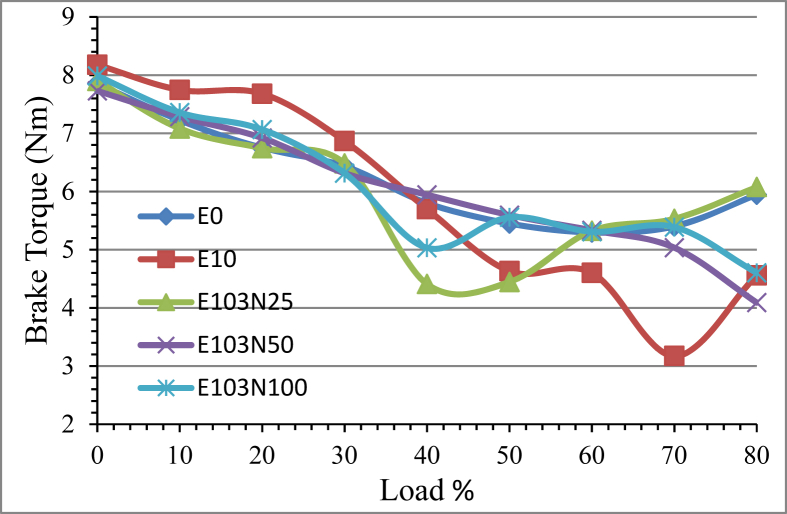
The effect of Nickel Zinc Iron Oxide (NiZnFe_2_O_4_) nanoparticles adding in E10 on engine brake torque.

As we change the load condition from minimum to about 60 percentages, the torque value slightly decreases for all the fuel samples in a closely similar fashion. The further load increase affected the torque value to increase. At low load condition the engine speed is high producing more number of power strokes resulting in high torque. During intermediate engine speed the torque reduces due to ignition delay and short ignition time. When the ethanol added emulsion fuel is compared with pure diesel the average torque value showed reduction E10 performed with higher torque at low load condition but at higher load condition the torque reduces drastically. This is because of the low calorific value and cetane number of the emulsified fuel. The adding of Nickel Zinc Iron Oxide (NiZnFe_2_O_4_) in a quantity of 25ppm, 50ppm and 100ppm increased the torque value as shown in Figure 8. Compared to E10 emulsion fuel, the nanoparticle added fuel samples produced a better torque output with increase of 3.5%, 3.03% and 3.53% for the fuel samples E103N25, E103N50 and E103N100 correspondingly.

#### 3.2.3 Brake power

**Figure 9:**
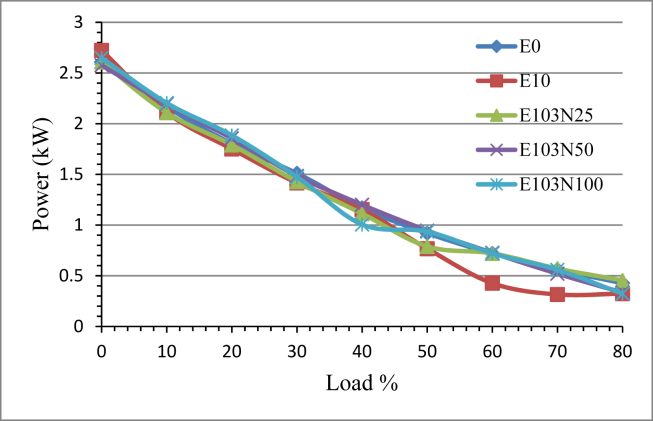
The effect of Nickel Zinc Iron Oxide (NiZnFe_2_O_4_) nanoparticles adding in E10 on engine Power.

The power output of the engine decreases as the load increases for all the fuel samples. Figure 9 shows the effect of Nickel Zinc Iron Oxide (NiZnFe_2_O_4_) nanoparticles on the power output of the E10 emulsion fuel. The power output is increased due to the use of the nanoparticle. Nanoparticle improves the fuel combustion properties because of the catalytic action. In comparison with E10, the fuel samples E103N25, E103N50 and E103N100 have shown improvement in engine power output of 5.43, 7.74 and 6.97 % respectively.

### 3.3 Emission

#### 3.3.1 Unburned hydrocarbon

**Figure 10:**
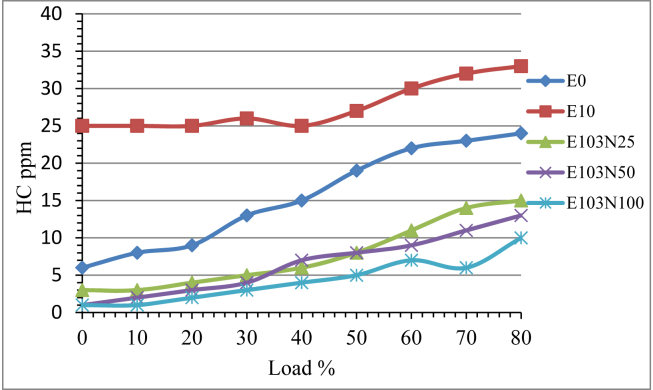
The effect of Nickel Zinc Iron Oxide (NiZnFe_2_O_4_) nanoparticles adding in E10 on HC emission.

The test result showed that the hydro carbon emission has increased as the engine load is increased. Ethanol is an oxygenated additive which enhances the combustion reaction and the adding greatly reduced the HC emission as shown in Figure 10. The addition of Ethanol in diesel fuel increased the HC emission by 78% because of the poor combustion resulted from low cetane number. To improve this problem the nanoparticle adding gave a promising result in reducing the HC emission. From the result it is observed that as the amount of nanoparticle increases the HC emission keeps on reducing. The HC reduction is recorded 50, 58 and 71% for E103N25, E103N50 and E103N100 fuel samples [25].

#### 3.3.2 Carbon monoxide

**Figure 11:**
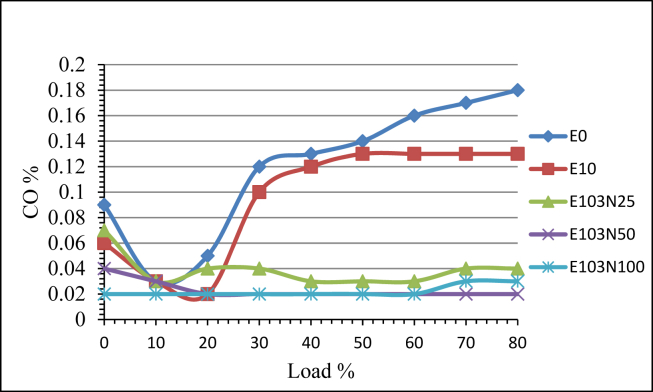
The effect of Nickel Zinc Iron Oxide (NiZnFe_2_O_4_) nanoparticles adding in E10 on CO emission.

Carbon monoxide (CO) emission is greatly influenced by the nanoparticles addition. At lower engine load the CO emission is higher and reduces as the load is increased further to nearly 20%. This is because at low load condition the engine runes at a high speed and this shorten the available time for complete combustion. The further load increment increases the fuel amount injected in to the cylinder and the cylinder pressure increment reduces the fuel spray efficiency making mixing of the fuel with air difficult and this makes the amount of CO emission to increase with load. E10 fuel reduced the CO emission by 20% due to high oxygen content. The addition of the nanoparticle further reduced the CO emission. Figure 11 shows that the fuel samples E103N25, E103N50, and E103N100, when compared to pure diesel, reduced CO emissions by 67, 80, and 81 percent, respectively, in accordance with [26].

#### 3.3.3 Carbon dioxide

**Figure 12:**
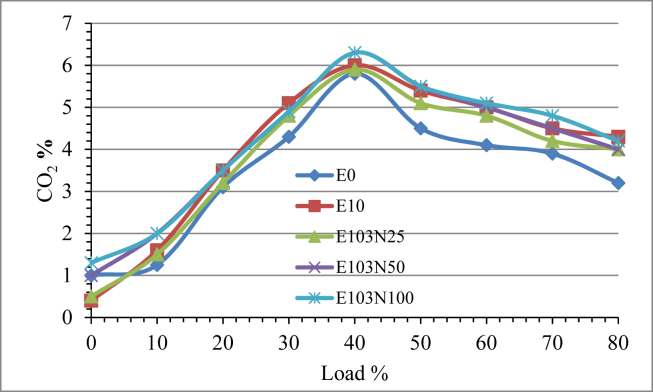
The effect of Nickel Zinc Iron Oxide (NiZnFe_2_O_4_) nanoparticles adding in E10 on CO_2_emission.

Carbon dioxide emission variation verses the load increment is shown in Figure 12. The addition of ethanol in diesel fuel increased CO_2_ emission compared to pure diesel fuel. The reason for this increment is because of ethanol diesel emulsion has high Oxygen content and this improves the combustion efficiency and oxidizes CO to CO_2_. When the effect of the nanoparticle is seen, as the amount of nanoparticle doped in the fuel increase the CO_2_ emission also increases. Compared to neat diesel (E0), E10, E103N25, E103N50 and E103N100 resulted in an average percentage increment of 14, 9, 16 and 19 respectively.

#### 3.3.4 Nitrogen oxide

**Figure 13:**
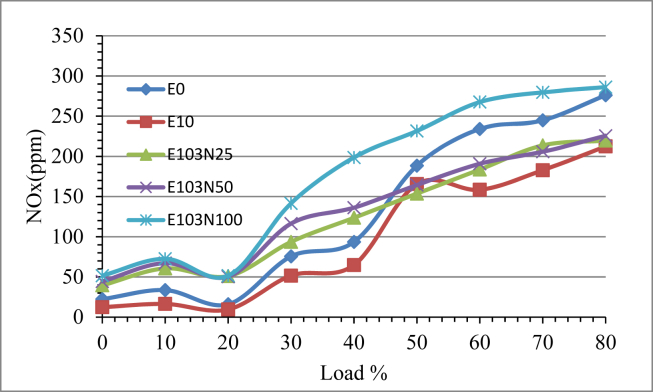
The effect of Nickel Zinc Iron Oxide (NiZnFe_2_O_4_) nanoparticles adding in E10 on NOx emission.

Figure 13 shows the comparative result of NO_X_ emission with load variation. From the graph it is seen the NO_X_ emission increases as the load on the engine increase due to at high engine load the combustion chamber pressure and temperature increases which creates favorable condition for the Nitrogen molecules (N_2_) to react with oxygen and form NO_X_. Ethanol emulsion fuel significantly reduced the NO_X_ emission compared to pure diesel fuel. Nanoparticle doping into the emulsion influenced the NO_X_ emission. As the amount of the nanoparticle added in to the fuel is increased, the NO_X_ emission also increased. The adding of nanoparticle in the fuel improves the combustion characteristics and this increases the cylinder temperature, as a result increasing the NO_X_ emission. The E10 fuel sample revealed a 26% drop, although the E103N25 and E102N50 fuel samples differed somewhat from the baseline diesel fuel. According to Gnanamoorthi et al. [26], an E103N100 gasoline sample had a 33 percent increase in NO_X_.

#### 3.3.5 Smoke

**Figure 14:**
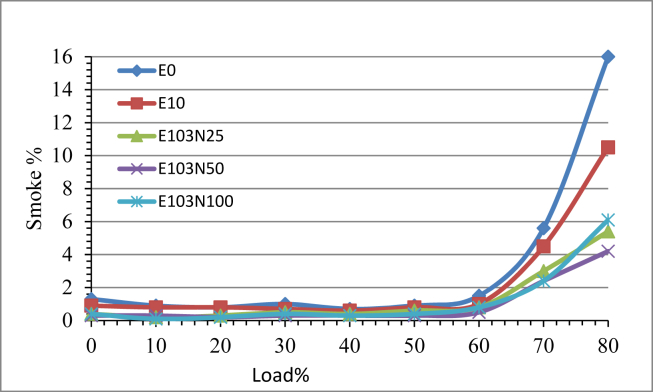
The effect of Nickel Zinc Iron Oxide (NiZnFe_2_O_4_) nanoparticles in E10 on Smoke emission.

The exhaust smoke density emitted from no load to 60 % load increment is less than 2% however the further load increment more than 60% makes the smoke emission to increase drastically for all the fuel samples. As shown in Figure 14 at higher condition it is clearly differentiated the influence of the nanoparticle on the smoke emission. Ethanol emulsion fuel reduced smoke emission compared to neat diesel. The addition of the nanoparticle further reduced the smoke emission and as the amount of the nanoparticle increase the intensity of exhaust smoke is reduced by 60, 69 and 61% for E103N25, E103N50 and E103N100 fuel samples respectively corresponding to Natarajan et al. [27].

. At high engine load the engines use rich mixture and this makes some of the fuel droplets not to react with oxygen and may escape in the form of Unburned or partially burned fuel which is the major constituents of exhaust smoke.

### 3.4 Benefits of this study

This research serves a variety of entities, including countries, organizations, and citizens. Countries with no or limited petroleum resources gain from lowering the amount of hard currency spent on fuel imports. Molasses is a byproduct of sugar processing that is produced by sugar factories. This by-product is increasingly being used to make ethanol. However, many sugar businesses do not use molasses properly for ethanol generation. The production of ethanol from the byproduct benefits the sugar industry. Ethanol might also be made from corn, sugar beets, and other agricultural feed stalks, therefore the research is beneficial to agricultural businesses and individuals. Citizens will have more job prospects as a result of the expanded ethanol manufacturing plants, which will help to stabilize the public economy. The entire community benefits due to the pollution reduction from the diesel engines.

## 4 Conclusion and recommendation

### 4.1 Conclusion

The stability of ethanol diesel emulsion is highly influenced by the amount of ethanol content which increases with the increase in the amount of ethanol.

The performance and emission characteristics of ethanol diesel emulsion fuel with adding Nickel Zinc Iron Oxide (NiZnFe_2_O_4_) nanoparticle is tested on attest rig of single cylinder four stroke diesel engines. From the test result, the following results are drawn.

The amounts of ethanol in ethanol diesel emulsion fuel greatly in fluencies the fuel consumption of an engine. The test result showed 27.17, 34.39 and 37.87 % bsfc increment for E5, E10 and E15 fuel samples respectively.

The presence of the nanoparticle in the fuel reduced the fuel consumption by 16.79, 27.86 and 27.48% for E103N25, E103N50 and E103N100 respectively compared with E10 without nanoparticle addition.

Brake torque increment of 3.5%, 3.03% and 3.53% for the fuel samples E103N25, E103N50 and E103N100 respectively due to the nanoparticle addition compared to E10. The power output is also increased by 5.43, 7.74 and 6.97 % for the same fuel samples respectively.

The addition of the nanoparticle gave tremendous reduction improvement in HC and CO emission. From the emission test result The HC emission reduction is recorded 50, 58 and 71% for E103N25, E103N50 and E103N100 fuel samples. CO emission is also reduced by 20% due to the addition of ethanol for E10 fuel and 67, 80 and 81% for E103N25, E103N50 and E103N100 fuel samples respectively.

Ethanol diesel emulsion NO_X_ emission result gave a reduction of 26 % but after the nanoparticle have been added the NO_X_ emission started to increase with the ppm increment of the nanopaticle. The highest increment is recorded by the fuel sample E103N100 which is 33% more compared to pure diesel fuel. Smoke opacity percentage is reduced with the use of ethanol emulsified fuel compared to pure diesel. More over the addition of the nanoparticle further reduced the smoke emission level. Due to the use of ethanol emulsion the reduction percentage is 28%, and a reduction of 59, 69 and 61% is recorded for the fuel samples E103N25, E103N50 and E103N100 respectively. The test result showed the adding of nanoparticle reduces smoke emission upto a certain amount and further increment of the additive increases the smoke level and the optimum result is seen to be 69 % for the fuel sample E103N50.

Considering the performance and emission characteristics of the prepared sample fuels E103N50, the one with 10% ethanol and 50ppm Nickel Zinc Iron Oxide (NiZnFe_2_O_4_) nano particle additive is more suitable than the other samples.

### 4.2 Recommendation

The authors recommend researchers to focus and conduct additional research as an extension of the currently produced research results. It is believed that the below list of areas are recommended to work in the area to promote ethanol diesel emulsion fuel and nanoparticle additives to its implementation stage in CI engine.

1. The effect of ethanol diesel emulsion fuel on the moving engine parts wear such as engine cylinder, piston, piston ring bearings valve and valve guide shall be seen in the future.
2. The influence of ethanol diesel emulsion fuel on the characteristics of engine lubricating oil shall be studied how it affects its physiochemical properties and determine the period of oil change interval.
3. The cost analysis of NiZnFe_2_O_4_ nanoparticle added fuels and its economic feasibility shall be studied.
4. The impact of NiZnFe_2_O_4_ nanoparticle on human health and environment shall be seen before marketing the fuel.
5. The design of mixing mechanism to maintain the stability of the emulsion during fuel storage and long time vehicle parking shall be studied in the future.
6. The fuel thank of the machine operated with ethanol diesel emulsion should be tight seal to protect the escaping of the ethanol by evaporation.

## Declarations

### Author contribution statement

Deresse Firew: Conceived and designed the experiments; Performed the experiments; Analyzed and interpreted the data; Contributed reagents, materials, analysis tools or data; Wrote the paper.

Ramesh Babu Nallamothu, Getachew Alemayehu: Conceived and designed the experiments; Performed the experiments; Contributed reagents, materials, analysis tools or data.

Rajendiran Gopal: Analyzed and interpreted the data.

### Funding statement

This research did not receive any specific grant from funding agencies in the public, commercial, or not-for-profit sectors.

### Data availability statement

Data will be made available on request.

### Declaration of interest's statement

The authors declare no conflict of interest.

### Additional information

No additional information is available for this paper.

## References

[1] Yu X., Sandhu N.S., Yang Z., Zheng M. (2020). Suitability of energy sources for automotive application – a review. Appl. Energy.

[2] Kumar N., Varun, Chauhan S.R. (2013). Performance and emission characteristics of biodiesel from different origins. Renew. Sustain. Energy Rev..

[3] Prabakaran B., Anurag U. (2016). Experimental investigation into effects of addition of zinc oxide on performance, combustion and emission characteristics of diesel-biodiesel-ethanol blends in CI engine. Alex. Eng. J..

[4] Bharathiraja M., Venkatachalam R., Senthilmurugan V. (2019). Performance, emission, energy and exergy analyses of gasoline fumigated DI diesel engine. J. Therm. Anal. Calorim..

[5] Liu Haifeng, Hu Bin, Jin Chao (2016). Effects of different alcohols additives on solubility of hydrous ethanol/diesel fuel blends. Fuel.

[6] Vasistha Vishal, Bharj Rabinder Singh (2019). Hydrous ethanol–diesel–Al2O3 nanoemulsified fuel characterization, stability, and corrosion effect. Energy Fuels.

[7] Syed Aalam C. (2020). Investigation on the combustion and emission characteristics of CRDI diesel engine fuelled with nano Al2O3 and Fe3O4 particles blended biodiesel. Mater. Today Proc..

[8] Yakasai F., Jaafar M.Z., Bandyopadhyay S., Agi A., Sidek M.A. (2021). Application of iron oxide nanoparticles in oil recovery – a critical review of the properties, formulation, recent advances and prospects. J. Petrol. Sci. Eng..

[9] Amanullah Md., Al-Tahini, Ashraf M. (2009).

[10] Agarwal S., Khan S. (2019). Effect of various nanoadditives on the performance and emission characteristics of a diesel engine fuelled with jojoba biodiesel–diesel blends. Plant Sci. Today.

[11] Khond V.W., Kriplani V.M. (2016). Effect of nanofluid additives on performances and emissions of emulsified diesel and biodiesel fueled stationary CI engine. Renew. Sustain. Energy Rev..

[12] Sajith V., Sobhan C.B., Peterson G.P. (2010). Experimental investigations on the effects of cerium oxide nanoparticle fuel additives on biodiesel. Adv. Mech. Eng..

[13] Venu Harish, Dhana Raju V., Subramani Lingesan (2019). Combined effect of influence of nano additives, combustion chamber geometry and injection timing in a DI diesel engine fuelled with ternary (diesel-biodiesel-ethanol) blends. Energy.

[14] Elango A., Karthikeyan S., Prathima A. (2014). An environmental effect of GSO methyl ester with ZnO additive fuelled marine engine. Ind. J. Geo-Mar. Sci..

[15] Hawi M., Elwardany A., Ismail M., Ahmed M. (2019). Experimental investigation on performance of a compression ignition engine fueled with waste cooking oil biodiesel–diesel blend enhanced with iron-doped cerium oxide nanoparticles. Energies.

[16] Balat M., Balat H. (2009). Recent trends in global production and utilization of bio-ethanol fuel. Appl. Energy.

[17] Kuszewski H., Jaworski A., Ustrzycki A., Lejda K., Balawender K., Woś P. (2017). Use of the constant volume combustion chamber to examine the properties of autoignition and derived cetane number of mixtures of diesel fuel and ethanol. Fuel.

[18] Soudagar Manzoore Elahi M., Nik-Ghazali Nik-Nazri, Abul Kalam Md., Badruddin I.A., Banapurmath N.R., Akram Naveed (2018). The effect of nano-additives in diesel-biodiesel fuel blends: a comprehensive review on stability, engine performance and emission characteristics. Energy Convers. Manag..

[19] Nanthagopal K., Kishna R.S., Atabani A.E., Al-Muhtaseb A.H., Kumar G., Ashok B. (2020). A compressive review on the effects of alcohols and nanoparticles as an oxygenated enhancer in compression ignition engine. Energy Convers. Manag..

[20] Debnath B.K., Saha U.K., Sahoo N. (2015). A comprehensive review on the application of emulsions as an alternative fuel for diesel engines. Renew. Sustain. Energy Rev..

[21] Dingcong W. (2002). A study of identifying the emulsion type of surfactant: volume balance value. J. Colloid Interface Sci..

[22] Mohammed S Gad, Abdel Razek Sayed M., Manu P.V., Jayaraj Simon (2021). Experimental investigations on diesel engine using alumina nanoparticle fuel additive. Adv. Mech. Eng..

[23] Ghannam M.T., Selim M.Y.E. (2009). Stability behavior of water-in-diesel fuel emulsion. Petroleum science and Technology. Petrol. Sci. Technol..

[24] Harne Madhavi S., Milind A., Pelagade, Ramakant S. (2017). Performance evaluation of CI engine using KaranjaBiodiesel as an alternative fuel. IJSRST.

[25] Gnanamoorthi V., Devaradjane G. (2015). Multi-zone modeling effect on combustion on DICI engine using ethanol diesel blend. Appl. Math. Sci..

[26] Gnanamoorthi V., Udhayakumar K., Devaradjane G. (2014). Experimental investigation of diesel – ethanol blend in DI diesel engine using preheating of intake air. Adv. Mater. Res..

[27] Natarajan S., Shankar S. Abhinav, Sundareswaran A.U. Meenakshi (2017). Early injected PCCI engine fuelled with bio ethanol and diesel blends – an experimental investigation. Energy Proc..

